# Syndecan-4 modulates the proliferation of neural cells and the formation of CaP axons during zebrafish embryonic neurogenesis

**DOI:** 10.1038/srep25300

**Published:** 2016-05-04

**Authors:** Ning Luo, Hongda Li, Bo Xiang, Liangjun Qiao, Jiao He, Yi Ji, Yuan Liu, Siying Li, Ran Lu, Yu Li, Wentong Meng, Yang Wu, Hong Xu, Xianming Mo

**Affiliations:** 1 Laboratory of Stem Cell Biology, State Key Laboratory of Biotherapy, West China Hospital, Sichuan University, and Collaborative Innovation Center for Biotherapy, Chengdu, 610041, China; 2 Department of Pediatric Surgery, State Key Laboratory of Biotherapy, West China Hospital, Sichuan University, and Collaborative Innovation Center for Biotherapy, Chengdu, 610041, China

## Abstract

Syndecan-4 (Syn4), a single-pass transmembrane heparin sulphate proteoglycan (HSPG), plays significant role in the formation of focal adhesions and interacts with many growth factors to regulate cell migration and neural induction. Here, we show the new roles of *syndecan-4*(*syn4*) in zebrafish embryonic neurogenesis. *Syn4* is broadly and dynamically expressed throughout the early stages of embryonic development. Knockdown of *syn4* increases the expression of the marker genes of multiple types of neural cells. The increased expression of the marker genes is resulted from excessive proliferation of the neural cells. In addition, disrupting *syn4* expression results in truncated and multiple aberrant branching of caudal primary (CaP) axons. Collectively, these data indicate that Syn4 suppresses the cellular proliferation during neurogenesis and is crucial for the formation of CaP axons during zebrafish embryogenesis.

Proteoglycans (PGs) are extracellular glycoproteins that contain sulphated glycosaminoglycan chains (GAG)^1^. Several studies have indicated that PGs play remarkable roles in regulating the interactions of cell surface molecules, such as the molecules mediating the cell-matrix, ligand-receptor and cell-cell interactions. They also function as co-regulators of many growth factors that are associated with neural fate, including FGF, HGF, Wnt, TGFβ and BMP^2^,^3^,^4^,^5^, indicating PGs act as important modulators during neural development. Syndecan-4 (Syn4) is a heparan sulphate PG in the Syndecan family^6^,^7^ and is composed of a variable extracellular domain, a single-span transmembrane domain and cytoplasmic domains that are highly conserved among the Syndecans^6^,^8^. As a unique family member, Syn4 is able to bind protein kinase Cα (PKCα) and phosphatidylinositol 4,5-bisphosphate (PIP_2_)^8^,^9^,^10^. Moreover, Syn4 interacts with integrin to be involved in the formation of focal adhesions and stress fibers^11^ and bind to chemokines^12^,^13^ to modulate the planar cell polarity^3^,^14^. During *Xenopus* and zebrafish embryonic development, *syn4* is expressed in the migrating neural crest and modulates the formation of polarized cell protrusion to control the directional migration^3^. Recent observations demonstrate that *syn4* is required for neural induction involving in FGF/ERK and PKCγ/Rac/JNK pathway^15^ and Wnt/PCP pathway to control the neural tube closure in mammalian embryos^14^. All these data show the important roles of *syn4* in embryonic neurogenesis. However, potential functions of *syn4* in the development of neural cells themselves have not been fully addressed.

In the present study, the roles of *syn4* during zebrafish embryonic neural development were addressed. The results showed that *syn4* was dynamically expressed throughout the early stages of zebrafish embryonic development. Knockdown of *syn4* promoted the generation of neural cells by enhancing the proliferation of neural cells. Furthermore, the length of CaP axon was severely reduced and the number of axonal branches was significantly increased as the expression of *syn4* was down-regulated. Taken together, these results indicate that *syn4* suppresses the proliferation of neural cells and is crucial for the formation of CaP axons.

## Results

### Spatio-temporal expressing patterns of *syn4* during zebrafish embryonic development

**Figure 1:**
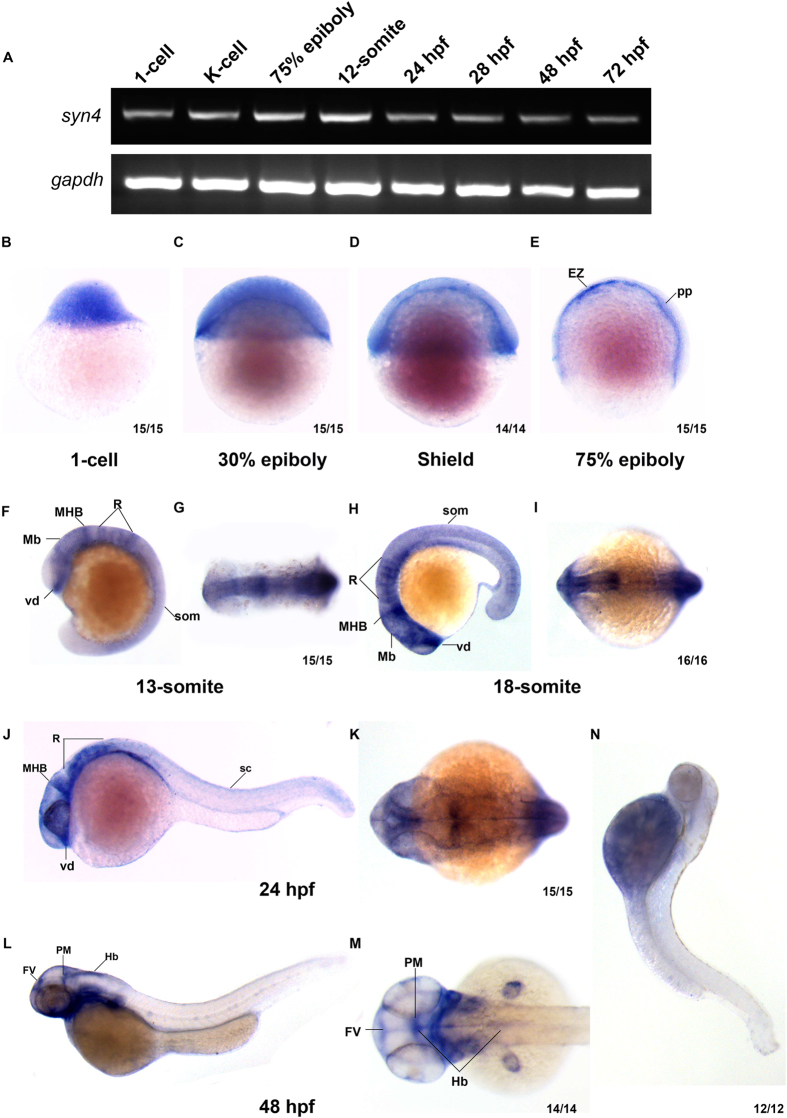
Syn4 expression pattern analysis in zebrafish. (**A**) sqRT-PCR reveals that *syn4* is expressed throughout embryonic developmental stages. (**B–M**) WISH shows the spatiotemporal expression pattern of *syn4*. (**B–D**) *Syn4* is ubiquitously distributed before the gastrulation. (**E**) In 75% epiboly, syn4 is expressed in the thin evacuation zone and prechordal plate. (**F–K**) *Syn4* is expressed in ventral diencephalon, midbrain, hind brain and neural crest during Segmentation Period. (**L,M**) In 48 hpf, *syn4* is restricted to the ventricular zone in the forebrain, posterior midbrain and hindbrain. (**N**) Negative control of *syn4* at 48 hpf. EZ: thin evacuation zone, FV: ventricular zone in the forebrain, Hb: hindbrain, Mb: midbrain, MHB: mid-hindbrain boundary, pp: prechordal plate, PM: posterior midbrain, R: rhombomeres, som: somites, sc: spinal cord, vd: ventral diencephalon. Lateral view, dorsal to the right in (**B–E**), dorsal view, anterior to the left in (**G,I,K,M**) lateral view, dorsal to the right and anterior to the top in (**F**), lateral view, dorsal to the top and anterior to the left in (**H,J,M**).

The zebrafish was used as a model organism to investigate the function of *syn4* in early embryonic development. The semi-quantitative reverse transcription-PCR (sqRT- PCR) and whole mount *in situ* hybridization (WISH) showed that *syn4* was expressed from 1-cell stage to 72 hours post fertilization (hpf). Notably, the *syn4* expressing level was higher at 12-somite stage. These results demonstrate that *syn4* is both maternally and zygotically expressed during zebrafish embryogenesis (Fig. 1A).

WISH analysis revealed that the spatial expression of *syn4* was dynamic during embryonic development. The *syn4* was ubiquitously distributed before the gastrulation (Fig. 1B,C). Then zygotic *syn4* mRNA highly expressed in the shield (Fig. 1D) and enriched in the thin evacuation zone and prechordal plate from the late gastrulation stage onward (Fig. 1E). Since the segmentation stage, *syn4* was predominantly expressed in rhombomere, ventral diencephalon, midbrain, hindbrain and neural crest till 24 hpf (Fig. 1F–K). Then *syn4* mRNA was restricted to the ventricular zone in the forebrain, posterior midbrain and hindbrain at 48 hpf (Fig. 1L,M). The detailed expressing patterns of *syn4* are consistent with previous observations in *Xenopus* embryos and zebrafish embryonic brains^3^,^14^,^16^. The results indicate that *syn4* has a highly dynamic expressing pattern and may play a role in the nervous development.

### *Syn4* regulates the migration of neural crest and muscle patterning

In the view of specific distribution of *syn4* in the neural tissues, we tried to investigate the function of *syn4* during zebrafish neural development. *Syn4* antisense morpholino oligonucleotide (*syn4* MO), which was previously reported to block the *syn4* translation^17^, was injected into embryos at the one-cell stage. As *syn4* is involved in neural crest (NC) migration^3^, we assessed whether the *syn4* MO we used were able to induce defective NC migration by analyzing the expression of the NC markers *crestin* and *sox10* and observed a strong inhibition of trunk NC migration in *syn4* MO-injected embryos at 18 somite stage (18hpf) (supplemental Fig. S1C–F). The results indicate that *syn4* MO is able to induce the defective NC migration as described t^3^,^14^. Given the role of Syn4 in muscle development^18^,^19^, we also performed the WISH to detect the expressing pattern of *myod*, which is a transcription factor that controls skeletal myogenesis^20^. The results showed that the expression of *myod* appeared abnormal curvature in the morphants (supplemental Fig. S2A–E). Fast and slow muscles in the trunk are stained with phalloidin, which recognizes actin fibers of both muscle types, at 18 somite stage (supplemental Fig. S3A–C”) and 26hpf (supplemental Fig. S3D–E”). Injection of *syn4* MO severely altered the morphology of muscle such that it appeared abnormally loose and wavy. Consistent with the previous observations^18^,^19^, the results indicate that *syn4* is involved in muscle development.

To test the efficiency and specificity of *syn4* MO, we co-injected the *syn4* MO with the pEGFP-N1-*syn4* recombinant plasmid that contained the *syn4* MO binding 5′-UTR sequences and evaluated the efficiency by analyzing the fluorescence intensity of *syn4*-GFP fusing protein. As expected, the green fluorescence intensity was severely reduced in the co-injected embryos (supplemental Fig. S1A,B). Co-injection of *syn4* mRNA without 5′-UTR *syn4* MO binding sequences was able to rescue the defective NC migration and muscle patterning (supplemental Fig. S1C–F). Altogether, these data demonstrate that the *syn4* MO is able efficiently to block *syn4* translation as described previously.

To further confirm the MO specificity, we design a series of truncated mutants based on a previous observations^21^ (supplemental Fig. S4A). All of the mutant mRNAs were injected into zebrafish embryos. The results demonstrated the mutant lacking the C2 domain in plasma membranes (*syn4-*ΔC2) showed the phenotypes highly similar to the ones induced by the *syn4* MO (supplemental Fig. S4B–J). The results clearly indicate that the ectopic expression *syn4* ΔC2 mRNA is able to disrupt the *syn4* functions in zebrafish embryos and the *syn4-*ΔC2 is disrupts syn4 function by acting as a dominant negative. Altogether, the results indicate the *syn4* MO and the mutant *syn4-*ΔC2 are able to reveal the specific roles of *syn4* involved in zebrafish embryonic development. The following results were mostly obtained from injection of *syn4* MO in zebrafish embryos. The mutant *syn4-*ΔC2 was used to confirm the specificity of the phenotypes caused by *syn4* MO in the following experiments.

### Knockdown of *syn4* expression increases neural progenitors

**Figure 2:**
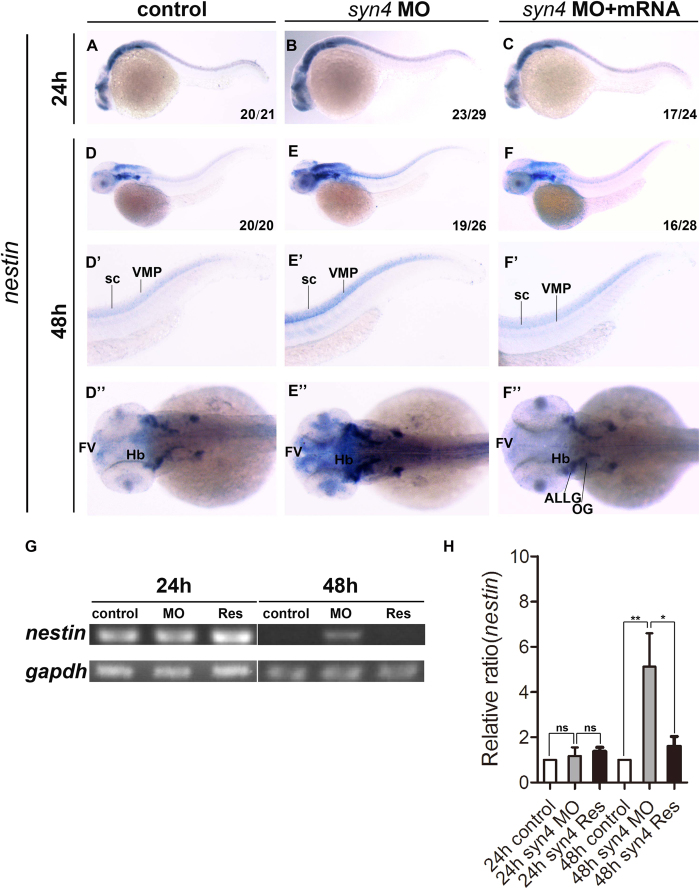
Knockdown of *syn4* expression up-regulated the expression of *nestin.* (**A–C**) Expression of *nestin* at 24 hpf, lateral view. (**D–F**) Expression of *nestin* at 48 hpf, lateral view. (**E**–**E**”) *Nestin* transcript level was increased significantly in *syn4* morphants, dorsal view. (**F**–**F**”) The defective phenotypes were rescued by co-injecting *syn4* mRNA. (**G**) sqRT-PCR showed the similar results. (**H**) Densitometric quantification of sqRT-PCR. The relative *nestin* expressing level is significantly higher in *syn4* morphants (mean ± s.e.m, n = 3, ***P < 0.001, **P < 0.01, *P < 0.05, ns = not significant, Student’s unpaired t-test). ALLG: anterior lateral line ganglion, FV: ventricular zone in forebrain, Hb: hindbrain, OG: octaval ganglion, sc: spinal cord, VMP: ventral motoneuron precursors.

Since *syn4* was expressed in neural tissues and required for neural crest migration, we then investigated whether *syn4* was involved in the embryonic neurogenesis. The effect of down-regulated *syn4* expression on neural stem cells and progenitor cells were examined by WISH with *nestin*^22^. The results showed that *nestin* expressing pattern was identical between the *syn4* MO-injected and the control embryos at 24 hpf (Fig. 2A,B). However, the expression level was significantly increased in the *syn4* morphants at 48 hpf (Fig. 2D–E”). Accordingly, sqRT-PCR showed the similar results (Fig. 2G,H). The *syn4* mRNA was able to rescue the defective phenotypes (Fig. 2F–F”) caused by the *syn*4 MO and the dominant negative mutant *syn4-*ΔC2 mRNA (supplemental Fig. S4B–G”). The results show that *syn4* negatively regulates *nestin* expression during embryonic neural development.

### Loss of *syn4* results in the increase of neuroglial cells

**Figure 3:**
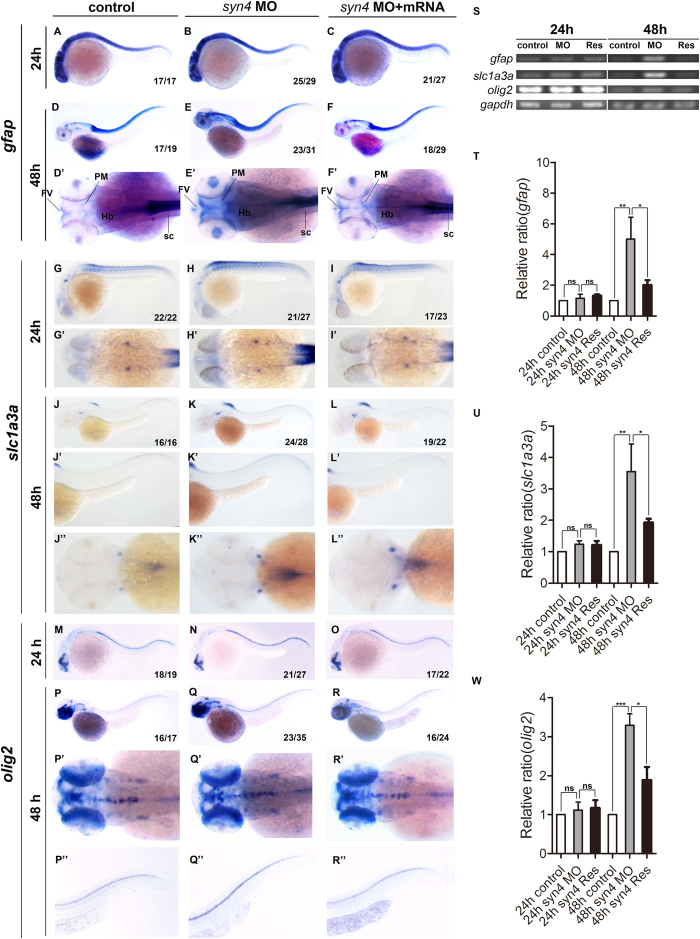
Loss of *syn4* increased neuroglial cells. (**A–R**”) WISH results of *gfap* (**A–F**”)*, slc1a3a* (**G**–**L**”) and *olig2* (**M–R**”) at 24 hpf and 48 hpf in zebrafish. (**D**–**F**’) The expression of *gfap*, (**J**–**L**”) *slc1a3a*, (**Q**–**R**”) *olig2* are higher in the *syn4* morphants than controls at 48 hpf. (**S**) sqRT-PCR analyzes of the transcript level of *gfap, slc1a3a* and *olig2*. (**T**–**W**) Densitometric quantification of sqRT-PCR. The *syn4* morphants shown a higher expression level of *gfap, slc1a3a* and *olig2* (mean ± s.e.m, n = 3, ***P < 0.001, **P < 0.01, *P < 0.05, ns = not significant, Student’s unpaired t-test). FV: ventricular zone in forebrain, Hb: hindbrain, PM: posterior midbrain, sc: spinal cord. Lateral views, dorsal to the top and anterior to the left in (**A–C**,**D–F**,**G–I**,**J–L’**,**M–O**,**P–R**,**P”–R”**), Dorsal view, anterior to the left in (**D’–F’**,**G’–I’**,**J”–L”**,**P’–R’**).

Since the neural stem cells/progenitor cells were regulated by Syn4, we were curious whether other neural cell types such as neuroglial cells were modulated by Syn4 as well. The expression of glial fibrillary acidic protein (*gfap*), an astrocyte marker, was not altered in the morphants and controls at 24 hpf (Fig. 3A,B), but up-regulated in the ventricular zone, especially in posterior midbrain and the spinal cord in the *syn4* morphants by 48 hpf (Fig. 3D–E’). The syn4 mRNA was able to abrogate the increased *gfap* expression (Fig. 3F,F’). Next, we tested the expression of *slc1a3a,* also known as *glast* that expresses in radial glia–astrocyte lineage^23^,^24^, in embryonic neural tissues. The results showed that there was little difference between the controls and the *syn4* morphants at 24 hpf (Fig. 3G–I’). The expressing levels of *slc1a3a* were obviously increased in dorsal spinal cord in the morphants at 48 hpf (Fig. 3J–K”). We then examined the expression of oligodendrocyte transcription factor 2 (*olig2*) in zebrafish embryos. *Olig2* is a marker of oligodendrocytes and motor neuron (MN) precursors^25^,^26^. Not surprisingly, the *olig2* expression was increased in the spinal cord in the *syn4* morphants at 48 hpf (Fig. 3P–R”). The sqRT-PCR analysis confirmed the altering patterns of the expressing levels of *gfap*, *slc1a3a,* and *olig2* in zebrafish embryos (Fig. 3S–W). Altogether, these data indicate that the neuroglial cells are increased when *syn4* expression is down-regulated.

### Knockdown of *syn4* expression promotes the generation of neurons

**Figure 4:**
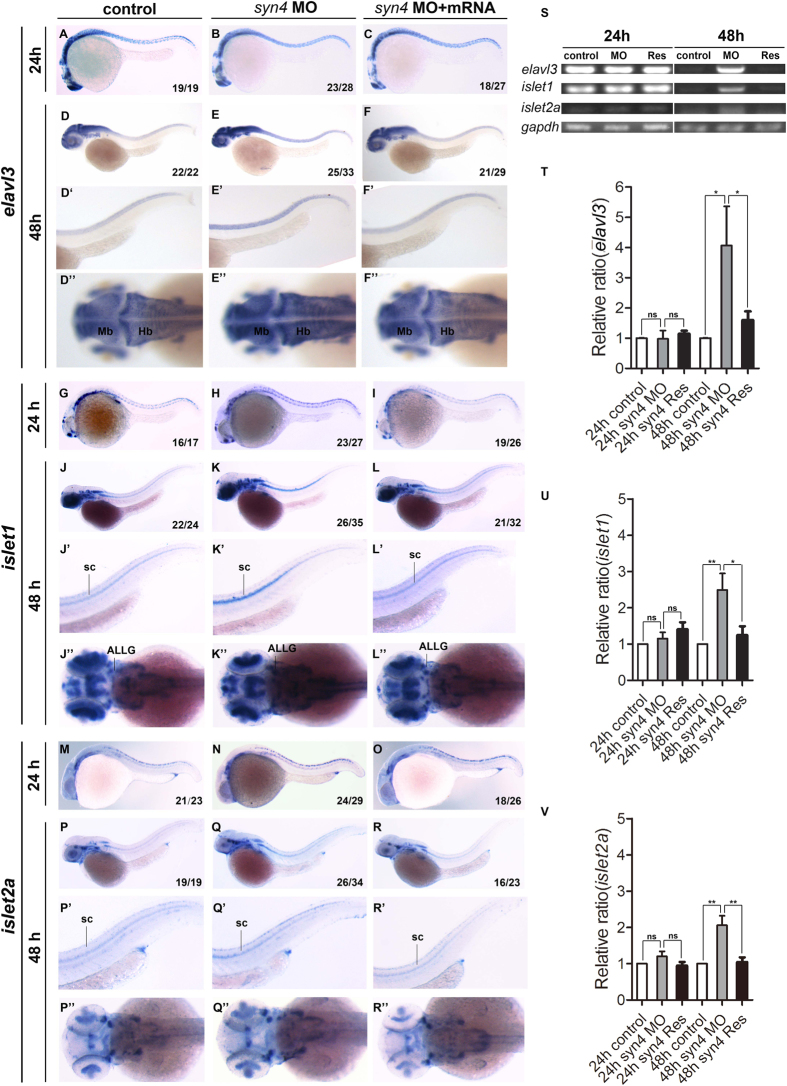
Knockdown of *syn4* expression promotes the generation of neurons. (**A**–**R”**) WISH results of *elavl3, islet1,* and *islet2a* at 24 hpf and 48 hpf in zebrafish. (**E**–**E**”) The expression of *elavl3*, (**K**–**K**”) *islet1*, (**Q**–**Q**”) *islet2a* in spinal cord were increased in *syn4* morphants at 48 hpf. co-injecting *syn4* mRNA was able to rescue the defects. (**S**) sqRT-PCR analyzes of the transcript level of *elavl3*, *islet1* and *islet2a.* (**T–V**) Densitometric quantification of sqRT-PCR. The *syn4* morphants shown a higher expression level of *elavl3*, *islet1* and *islet2a* (mean ± s.e.m, n = 3, ***P < 0.001, **P < 0.01, *P < 0.05, ns = not significant, Student’s unpaired t-test). ALLG: anterior lateral line ganglion, Hb: hindbrain, Mb: midbrain, sc: spinal cord. Lateral views, dorsal to the top and anterior to the left in (**A–F’,G–L’,M–R’**). Dorsal view, anterior to the left in (**D”–F”,J”–L”,P”–R”**).

Since *syn4* modulated the number of neural progenitors and neuroglial cells, we further tested whether *syn4* was involved in neuron specification. Thus, we examined the expression of *elavl3*, the earliest marker of pan-neuronal cells^27^, in zebrafish embryos. Knockdown of *syn4* didn’t alter the expression of *elavl3* at 24 hpf (Fig. 4A,B). However, injection of *syn4* MO resulted in a dramatic increased expressing levels of *elavl3* in spinal cord compared with the controls at 48 hpf (Fig. 4D–E”).

To test the formation of motor neurons in *syn4*-depleted embryos, we analyzed the expression of *islet1* and *islet2a*, which label primary motoneurons (PMNs), sensory Rohon-Beard neurons, and retina neurons. Rostral primary (RoP) and middle primary (MiP) MNs express *islet1* but not *islet2a*, while CaP and variable primary (VaP) MNs express *islet2*^28^. The *islet1* and *islet2a* expression were both increased in ventral spinal cord in the morphants at 48 hpf (Fig. 4J–K”). Co-injection of *syn4* mRNA was able to rescue the enhancement of *islet1* and *islet2a* expression induced by the *syn4* MO (Fig. 4J–L”). The statistical results of sqRT-PCR were consistent with the results of WISH (Fig. 4S–V).

To gain further insight into the functions of *syn4*, we also analyzed another member of Syndecan family in zebrafish, *syndecan2* (*syn2*) and found that *syn2* mRNA was unable to rescue the phenotypes caused by the *syn4* MO (supplemental Fig. S5A–F’). Taken together, the observations demonstrate that Syn4 modulates the embryonic development of neural stem cell/progenitors, neuronal glial cells, and neurons.

### The proliferation of neural cells is increased by down-regulation of the *syn4* expression

**Figure 5:**
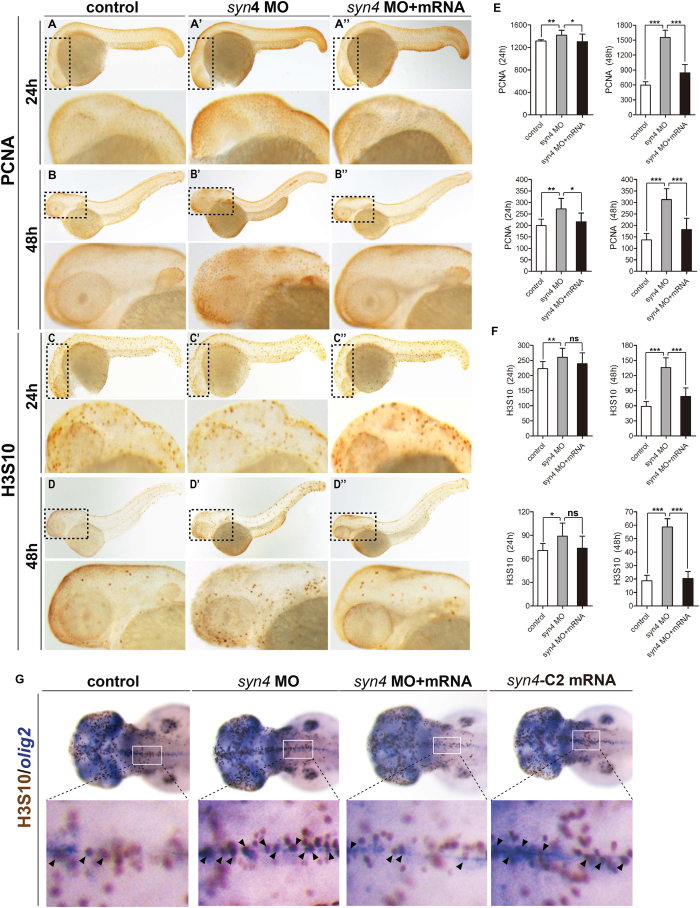
Whole-mount immunohistochemistry staining of PCNA and H3S10 in zebrafish embryos. (**A**–**D”**) Immunostaining of PCNA and H3S10 in 24 hpf and 48 hpf embryos, the box regions are magnified. (**E**,**F**) Quantification of PCNA-positive cells (**E**) and H3S10 -positive cells (**F**) in different treatment groups. Both PCNA positive cells and H3S10 positive cells are considerably increased in *syn4* morphants and the defects were partially rescued by co-injection with *syn4* mRNA. For each group, 18 embryos are scored. (mean ± s.e.m, n = 3, ***P < 0.001, **P < 0.01, *P < 0.05, ns = not significant, Student’s unpaired t-test). (**G**) The double WISH with H3S10 and *olig2*. Arrows label the H3S10 positive cells, the box regions are magnified. Lateral views, dorsal to the top and anterior to the left in (**A**–**D”**,**G**).

To reveal how the neural cells were regulated by the *syn4* expression, we performed immunohistochemistry staining of whole-mount embryos to detect the proliferating cell nuclear antigen (PCNA), which is a marker of cells with proliferative potential^29^. Statistical results revealed that the numbers of PCNA positive cells were increased in the *syn4* morphants at 48 hpf. The phenotypes were fully rescued by the *syn4* mRNA (Fig. 5A–B”). We also counted phospho-Histone H3 (Ser10), which is another marker to label the proliferative cells, positive cells in zebrafish embryos. The results showed that the number of phospho-Histone H3 (Ser10) positive cells was increased in the morphants at 48 hpf (Fig. 5C–D”). The increased staining cells were more obvious in zebrafish embryonic brains (Fig. 5E,F, supplemental Fig. S6A–C’). Examination of the embryo sections also showed that the phospho-Histone H3 (Ser10) positive cells were increased in the 48hpf zebrafish embryos that were disrupted the Syn4 function (supplemental Fig. S6D–G). In addition, we also performed Two-Color Whole-Mount Staining with DAB and BCIP/NBT to identify whether neural cells, including neuroglial cells and motor neurons, carried increasing signal of phospho-histone H3 in zebrafish embryos after disruption of syn4 function. The results showed that proliferation of all tested types of neural cells was up-regulated in the morphants at 48 hpf (Fig. 5G and data not shown). Taken together, our data indicate that Syn4 suppresses the proliferation of neural cells during zebrafish embryonic neurogenesis.

### *Syn4* is required for the formation of CaP axons

**Figure 6:**
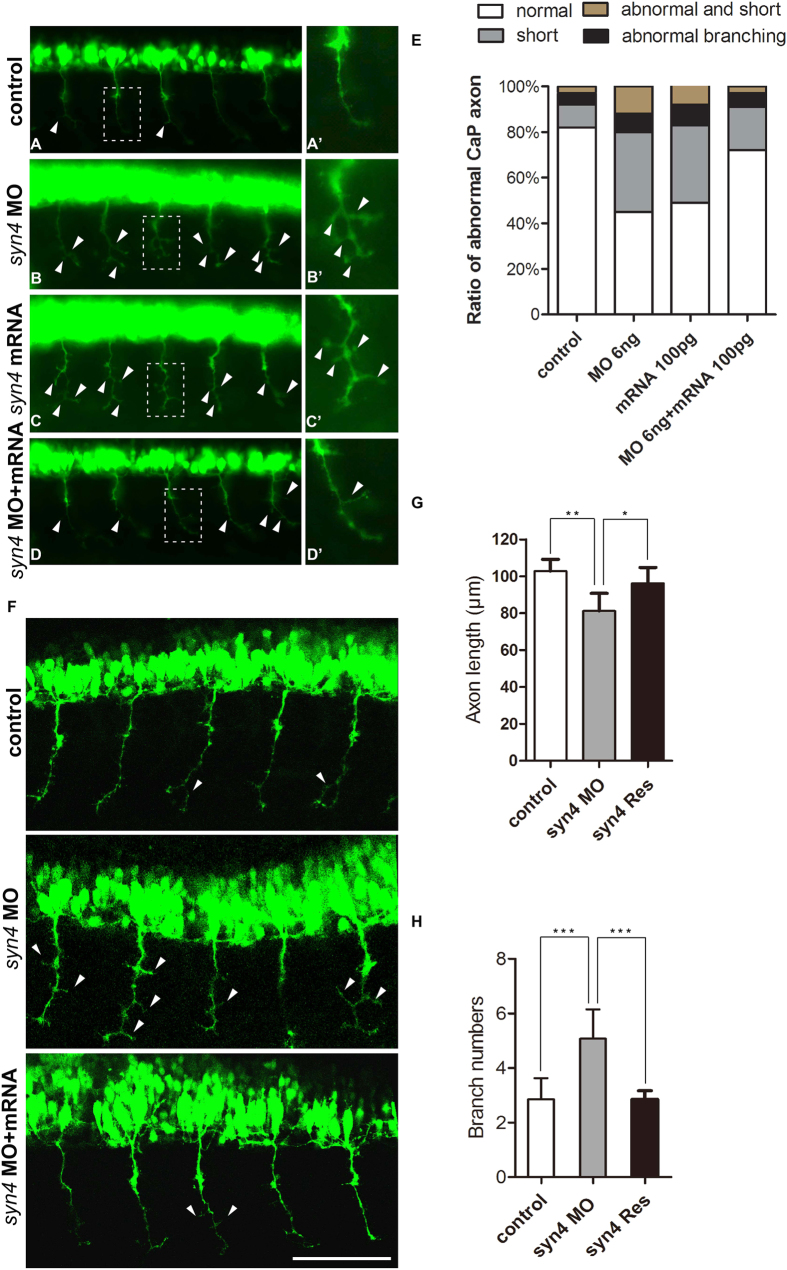
Syn4 is required for the CaP axons formation. (**A**–**D**’) Embryos at 26 hpf show PMNs in green fluorescence. (**B**,**B’**) *Syn4* morphants show shortened and branching CaP axons, (**C**,**C’**) *syn4* mRNA-injected embryos have same phenotypes as *syn4* morphants. (**D**,**D’**) Co-injection of *syn4* MO with *syn4* mRNA partially rescues CaP axon defects. (**E**) Statistical results of CaP axon outgrowth are shown in (**A**–**D’**). For each group 60 axons from 12 embryos are scored. (**F**) The CaP axons of control embryos and *syn4* morphants are photographed under confocal microscopy. (**G**) Statistical results of axon length in (**F–G**). The length of CaP axons is reduced in *syn4* morphants (**P < 0.01, t-test). (**H**) the branch number of CaP axons is increased (***P < 0.001, t-test) in *syn4* morphants. For each group 75 axons from 15 embryos are scored. The box regions are magnified and show in (**A’–D’**). Arrows indicate shortened CaP axons and abnormal branchs. Lateral views, dorsal to the top and anterior to the left in (**A–D’**,**F**). Scale bars: 100 μm.

As the expression of several markers of motor neuron was altered in the *syn4* MO injected embryos, we considered that *syn4* might be associated with motor neuron development. There are two distinct classes of spinal motor neurons: PMNs and secondary motor neurons (SMNs) in zebrafish^30^. The PMNs are classified into CaP, MiP, and RoP MNs^31^. These PMNs can be identified by their stereotypical cell body positions and axon projection patterns. Specially, the CaP MNs is easy to be observed as their locates in the middle of each spinal cord and axonal projection extends ventrally^32^. Therefore, we selected CaP MNs as the target objects in *Tg* [hb9: GFP]^ml2^ transgenic zebrafish. The the expression of islet1/2a was not altered before 48hpf (supplemental Fig. S2F–K), however, 56% *syn4*-MO injected embryos showed abnormal PMNs with multiple aberrant branching axons and truncated axons at 26 hpf of the zebrafish embryos (Fig. 6A–B’). To assess whether overexpression of *syn4* was able to disturb the outgrowth of CaP axons, we injected *syn4* mRNA at one-cell stage and quantitated the number of PMNs with defective phenotypes at 26 hpf. The result showed that 53% *syn4* mRNA-injected embryos had shortened CaP axons and/or abnormally branched axons (Fig. 6C). The length of CaP axon outgrowth in the morphants averaged 79.9 um, which was significantly reduced, compared to the control embryos’s CaP axons that averaged 100.2 um (Fig. 6F,G). In addition, the averaged branch number of per CaP axon in *syn4* MO-injected embryos was 4.71, while control embryos carried only 3.57 branches in average (Fig. 6F,H). The *syn4* mRNA co-injection was able to rescue the defective CaP axon morphology (Fig. 6D). These results indicate that accurate *syn4* expression is crucial.

## Discussion

Previous observations have shown that Syn4 is an element of the PCP non-canonical Wnt signaling pathways to regulate the neural tube closure and neural crest migration during *Xenopus* and zebrafish embryonic development^3^,^14^. Syn4 also modulates the neural induction through the ERK or PKC-dependent signaling pathways^15^. Here, the new role of *syn4* is revealed in our works. We identify that the neural cells are prominently increased after the inhibition of *syn4* during the early stage of zebrafish embryonic neurogenesis. Further evidences reveal that *syn4* inhibits the cell proliferation during the development of neural system and maintains motorneuron morphology.

The prior data have indicated that Syn4 has a positive effect on cell proliferation by regulating PKCα activation to mediate growth factor-stimulated proliferation in the wounded adult tissue and adult skeletal muscle cells^2^,^33^,^34^,^35^. Syn4 has negative effects on the cell proliferation in multiple tumor cell lines such as breast carcinoma and glioblastoma cells. In the tumor cells, the ability of Syn4 to bind to the fibronectin is competitively blocked by tenascin-C in integrin signaling, resulting in the proliferation of tumor cells^36^. Since Syn4 is indispensable to the cell spreading by interaction with the HepII site in fibronectin, disrupting the interaction between Syn4 and FNIII13 results in cell spreading obstacle^37^,^38^. The data suggest that there exists a mechanism that fibronectin signaling attenuates cell proliferation by Syn4^36^. In consistent with the results obtained from the tumor cell lines, we find that Syn4 inhibits the proliferation of neural cells. Therefore, further work is required to determine whether fibronectin is involved in the inhibition of neural proliferation during embryonic neural development.

The extension of axons and dendrites to form stereotypic neuronal connections is crucial to neural development. It has been reported that the biosynthesis of heparan sulfate (HS) is required for normal axon elongation and branching in animals, including mouse, C. elegans and zebrafish. HSPGs, the carrier proteins of HS, are involved in properly axon architecture. *Syndecan-3* and *glypican-2* are shown to be required for axon guidance^5^,^39^. However, little is known about the role of *syn4* in the architecture of axon. We identify that perturbed expression of *syn4* results in aberrant elongation and abnormal additional branching of CaP axons. The C2 domain of Syn4 is essential for the function of *syn4* in the formation of CaP axons. These evidences is similar to the phenotype that defects in the extension and targeting of axons caused by abnormal FGF signaling, suggesting that Syn4 may regulate the axon architecture via the FGF signaling in the zebrafish^5^,^40^,^41^. Future experiments are required to explore the factors that are combined with *syn4* in regulating CaP axons formation.

Our results show that muscle patterning is altered and the morphology of muscle severely altered in the *syn4* morphants since 18hpf. The axons of motor neuron show aberrant elongation and additional branching in the morphants at 26hpf. In view of the axon of primary motor neuron start to project to the myotomes at 18hpf, while the myotomes have formed before the time point^42^,^43^. Our results suggest that the early defective muscle patterning induced by knockdown of Syn4 functions may be independent of the proliferation of the neural cells. The results also suggest that the early defective muscle patterning may contribute to defective axon branching and length in the *syn4* morphants. However, further experiments are required to address the question. *Syn2* and *syn4* have a high degree of structural similarity^44^. However, we identify that overexpression of *syn2* is unable to rescue the phenotypes caused by knockdown of Syn4 functions in present works. The results indicate that Syn4 specifically modulate the proliferation in neural cells and axon patterning of motor neurons and are consistent with previous observations that Syn2 and Syn4 are engaged in a number of distinct developmental processes^3^,^45^,^46^.

## Methods

### Zebrafish maintenance and strains

All experiments performed on zebrafish (Danio rerio) were according to standard procedures^47^. Embryos were staged as described and raised at 28.5 °C in the same system as adults (Aquatic Ecosystems)^48^. The Tg[hb9:GFP]^ml2^ transgenic lines^49^ and AB strains were used in our studies.

All zebrafish experiments were performed in accordance with the guidelines of the animal ethical committee of West China Hospital. All experimental protocols were proved by the Animal Ethical Committee, West China Hospital, Sichuan University.

### *Syn2/4* cloning

Syn2/4 cDNA was amplified from zebrafish embryos using the primers shown in the supplemental Table S1 and subcloned into the pGEM-T easy vector to synthetize antisense probes for *in situ* hybridization. The coding region of syn2/4 was subcloned into the pcDNA3.1+ vector for mRNA synthesis. The *syn4* primer1 resultant PCR products (with 5′-UTR sequence) were ligated into pEGFP-N1 vector.

### Construction of *syn4* mutants

Five mutants of zebrafish *syn4* were designed based on a previous observations^21^ and generated by PCR. The primers were shown in the supplemental Table S1. The C-terminal domain deletion mutants included ΔC1, ΔC2, ΔV and ΔC1–2. The N-terminal domain deletion mutant ΔEc lacks the extracellular domain.

### Total RNA isolation and Semi-quantitative Reverse transcription-PCR (sqRT-PCR) and Statistical analysis

Total RNAs of an approximate 100 embryos at different stages, including 1-cell, K-cell, 75% epiboly, 12-somite, 24 h, 28 h, 48 h, 72 h post-fertilization (hpf) were isolated. Each reaction used 20μl total RNA with the Prime Script Reverse-transcription PCR kit (TaKaRa DRR014A). The resultant cDNA were used as the templates for PCR. The primer pairs used for sqRT-PCR analysis were shown in the supplementary (supplemental Table S1). Statistical analysis was performed using Student’s unpaired t-test. Differences were considered significant for p < 0.05.

### *In vitro* transcription of Antisense RNA probes and mRNAs

Antisense RNA probes for *in situ* hybridization were synthesized *in vitro* following manufacturer’s instructions of Riboprobe system kit (Promega). Plasmid was linearized and the probes *syn4, elavl3, gfap, islet1, islet2a, olig2, slc1a3a* and *nestin* were synthesized. Capped *syn4* mutant mRNA and full-length mRNA that lacked the morpholino sequence were synthesized using the T7 Mmessagem MACHINE kit (Ambion).

### Antisense morpholino and mRNA or plasmid injections

Antisense morpholino oligonucleotides (MO) against *syn4*: TGAGGTAAACTTTCAACAT CTTCTC were purchased from Gene Tools (Philomath, OR, USA) and used as previously described^17^. The mRNAs and MO were injected into the yolk and the plasmid was injected into the cell at the one cell stage. Unless stated otherwise, a volume of 1 nl was injected into embryos with the concentration of 6 ng/nl of MO, 100–150 ng/ul of mRNA and 100–200 ng/ul of plasmid.

### Whole-mount *in situ* hybridization and histology

Whole-mount *in situ* hybridization (WISH) or the double WISH with DAB and NBT/BCIP was carried out as previously described^50^. The antisense RNA probe was synthesized from the relative cDNA with a digoxygenin (DIG) RNA labeling kit (Roche). In brief, embryos were permeabilized with Proteinase K (10 mg/mL, Promega) for 2 minutes/24 hpf or 30 minutes/48 hpf. Hybridization was done overnight at 65 °C with the DIG-labeled antisense probes. After washed at 65 °C with formamide and SSCT concentration gradient (50% formamide, 2*SSCT to 0.2*SSCT), restore to the room temperature and washed again (0.1*SSCT to 0.05*SSCT), DAB staining followed NBT/BCIP (Roche) staining was performed according to the manufacturer’s instructions. The following probes were used: *syn4, crestin, elavl3, slc1a3a, gfap, islet1, islet2a, olig2, sox10* and *nestin*.

### Immunohistochemistry Staining of Whole-mount Embryos and Statistical analysis

Immunohistochemistry was performed using these sections as previously described^51^, the following primary antibodies were used: mouse anti-PCNA (Calbiochem 1:500) and rabbit anti-phospho-Histone H3 (Ser10) (Millipore 1:500). We counted the positive cells at the brain and the whole body of each fish, and calculate the average to compare the difference among groups. Statistical analysis was performed using Student’s unpaired t-test. Differences were considered significant for p < 0.05.

### Immunofluorescence staining

Immunofluorescence staining was performed as previously described^52^ using the following antibodies: rabbit anti-phospho-Histone H3 (Ser10) (Millipore 1:200), phalloidin (Sigma 1:1000). In brief, Zebrafish embryos were fixed in 4% paraformaldehyde at 4 °C overnight, washed three times in PBS for 5 min each wash, then incubated in acetone at −20 °C for 7 min and washed three times in PBS for 5 min each wash. Blocking the embryos with 5% BSA and dilute the antibodies with blocking solution used for immunostaining at 4 °C overnight.

For cross-section samples, embryos were equilibrated in 30% sucrose prepared in PBS and embedded in optimal cutting temperature compound at −20 °C. Sections with a thickness of 8–10 um.

## Additional Information

**How to cite this article**: Luo, N. *et al.* Syndecan-4 modulates the proliferation of neural cells and the formation of CaP axons during zebrafish embryonic neurogenesis. *Sci. Rep.* **6**, 25300; doi: 10.1038/srep25300 (2016).

## Supplementary Material

Supplementary Information
