# Supplementary material for: Syndecan-4 modulates the proliferation of neural cells and the formation of CaP axons during zebrafish embryonic neurogenesis
Source: Sci Rep. 2016 May 4;6:25300. doi: 10.1038/srep25300 (PMC4855150; doi:10.1038/srep25300)
Supplement: Supplementary Information [file srep25300-s1.pdf]

Syndecan-4 modulates the proliferation of neural cells and the formation of CaP axons during zebrafish embryonic neurogenesis

Ning Luo[#], Hongda Li [#], Bo Xiang, Liangjun Qiao, Jiao He, Yi Ji, Yuan Liu, Siying Li, Ran Lu, Yu Li, Wentong Meng, Yang Wu, Hong Xu, Xianming Mo*

Supplementary figure legend

Figure S1. *Syn4* is required for the migration of neural crest.

(A, B): Knockdown efficiency of *syn4* MO at 9 hpf. (A): Zebrafish embryos were injected with 30 pg of pEGFP-N1-*syn4* recombinant plasmid containing the MO-binding site. (B): Green fluorescence was totally abolished by co-injection with 6 ng *syn4* MO. (C-D”): WISH assays of *crestin* (C-C”), *sox10* (D-D”) at 18 somites stage. Arrows indicate the expression of crestin/sox10 in the morphants are inhibited. (E, F): Quantification of phenotypes produced by *syn4* morphants at 18 somites stage, with or without *syn4* mRNA. Lateral views, dorsal to the top and anterior to the left in C-D’’.

Figure S2. Knockdown syn4 disrupt the muscle patterning of zebrafish.

In situ hybridization for indicated genes. (A-D”) WISH assays of *myod* at 18 somites stage, white bars marks vertical. The morphants and syn4-C2 mRNA injected fishs appeared abnormal curvature. (E): Quantification of phenotypes at 18 somites stage. (F-K): Expression pattern of *islet1/islet2a* in different embryos at 18 somites stage.

Figure S3. Knockdown syn4 disrupt the muscle patterning of zaebrafish.

(A-C”) Fast and slow muscle structures in Tg(Hb9:GFP line) at 18s. Fast and slow muscles in the trunk are stained with phalloidin, which recognizes actin fibers of both muscle types, PMNs in green fluorescence. Phalloidin labeling revealed an impaired muscle structure in morphants. (D-F”) muscle structures in Tg(Hb9:GFP line) at 26 dpf. Phalloidin labeling revealed the fibers are abnormally thick and wavy in morphants. The box regions were magnified.

Figure S4. The C2 domain of *syn4* is crucial for the proliferation of neural cells and the formation of CaP axons

(A): Schematic representation of Syndecan 4 constructs. (B-G”): Expression pattern of *nestin* in different embroyos at 24 hpf (B-D) and 48 hpf (E-G”). (H): Embryos at 26 hpf showing PMNs in green fluorescence. The box regions were magnified. Arrows indicate shortened CaP axons and abnormal branchs. (I): Statistical results of relative ratio of nestin expression level in (E-G”). (J): Quantification of CaP axon branching in (H), the number of axonal branches was significantly higher in syn4 morphants and syn4-ΔC2 mRNA injected embryos than in controls. For each group 84 axons from 14 embryos were scored (mean $\pm$s.e.m, n=3, ***$P<0.001$, **$P<0.01$, *$P<0.05$, ns= not significant, t-test). Hb: hindbrain, sc: spinal cord.

Figure S5. Overexpression of syn2 is unable to rescue the phenotypes caused by knockdown of Syn4 functions

In situ hybridization for indicated genes. (A-F’): WISH assays of *gfap* and *elavl3* in different

embryos at 48 hpf. The higher expression level of *gfap*/*elavl3* in morphants at 48 hpf couldn't be rescued by co-injection with syn2 mRNA. FV: ventricular zone in the forebrain, Hb: hindbrain, Mb: midbrain.

Figure S6. Immunofluorescence staining of H3S10 in zebrafish embryos

(A-C'): Immunostaining of H3S10 in 48 hpf embryos. (D-F"): Transverse sections of forebrain (Top), midbrain (middle) and hindbrain (below) of different embryos immunostained for H3S10 (green).G: Quantification of H3S10 -positive cells. For each group, 6 embryos are scored (mean ± s.e.m, n=3, ***$P<0.001$, **$P<0.01$, *$P<0.05$, ns= not significant, Student's unpaired t-test). e: eye, Fb: forebrain, Hb: hindbrain, Mb: midbrain, t: tectum. The dotted boxes mark the brain regions.

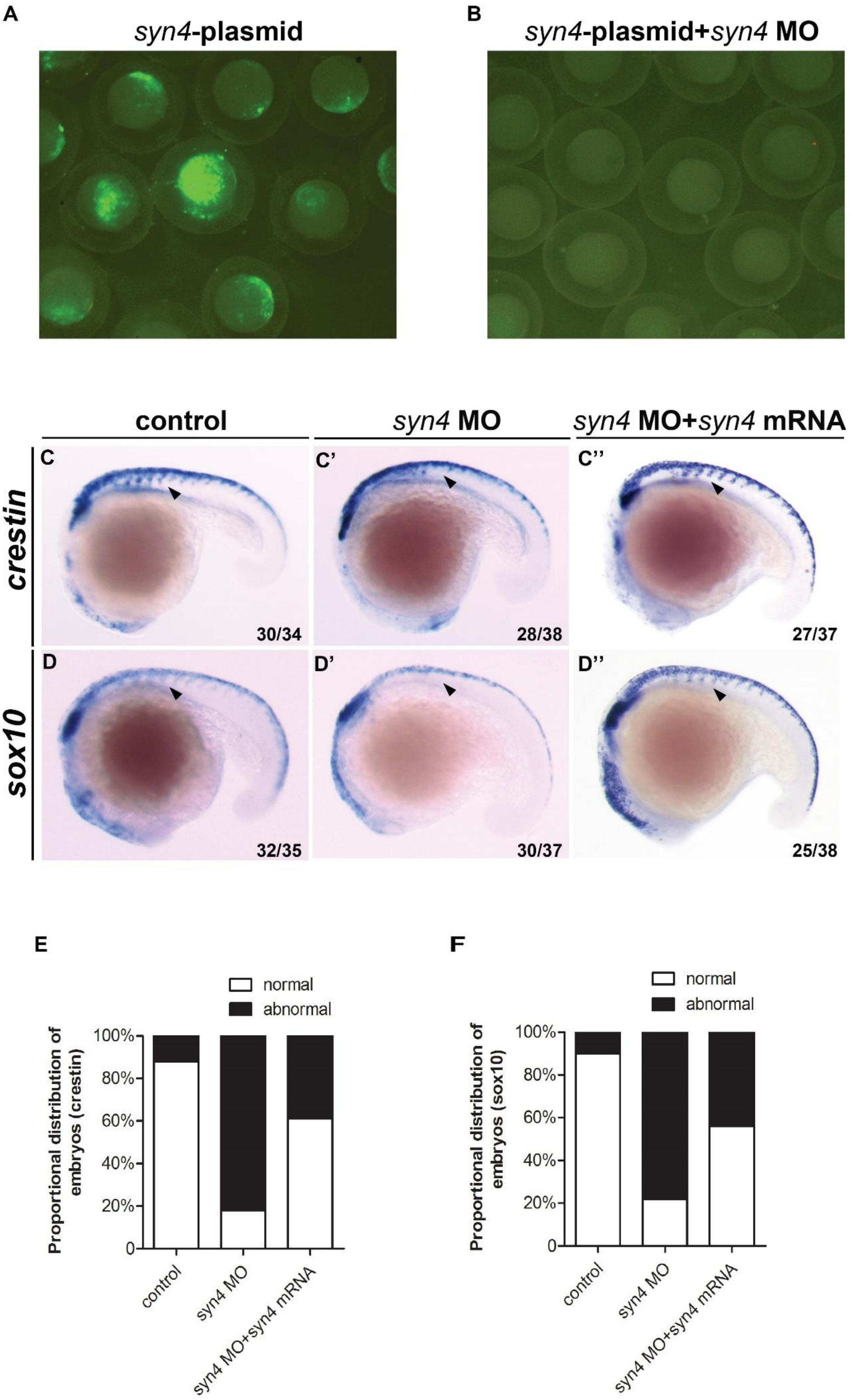

A
syn4-plasmid
B
syn4-plasmid+syn4 MO
control
syn4 MO
syn4 MO+syn4 mRNA
crestin
sox10
C
C'
C''
30/34
28/38
27/37
D
D'
D''
32/35
30/37
25/38
E
normal
abnormal
Proportional distribution of embryos (crestin)
100%
80%
60%
40%
20%
0
control
syn4 MO
syn4 MO+syn4 mRNA
F
normal
abnormal
Proportional distribution of embryos (sox10)
100%
80%
60%
40%
20%
0
control
syn4 MO
syn4 MO+syn4 mRNA

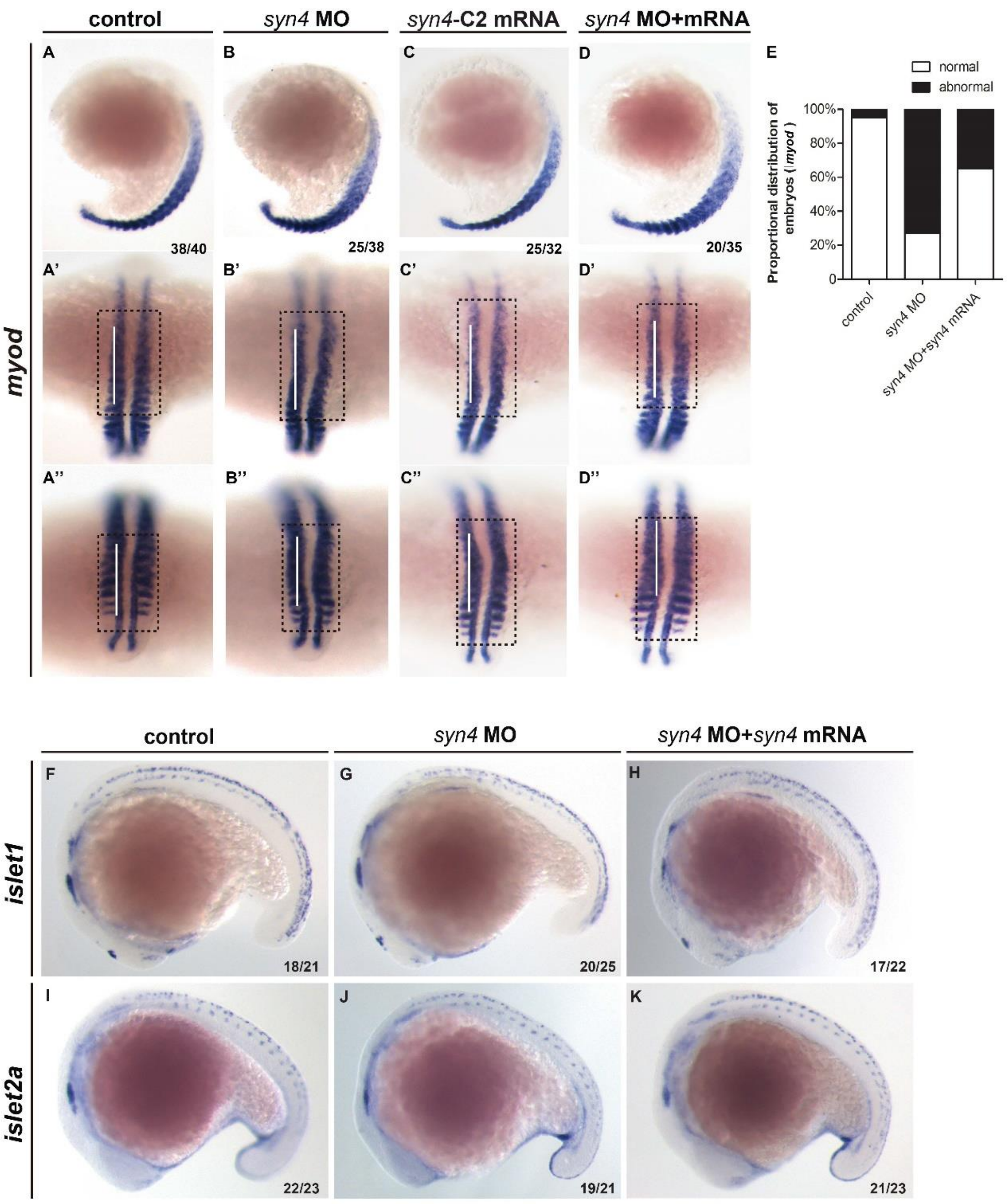

control
syn4 MO
syn4-C2 mRNA
syn4 MO+mRNA
myod
A
B
C
D
38/40
25/38
25/32
20/35
A'
B'
C'
D'
A''
B''
C''
D''
E
normal
abnormal
Proportional distribution of embryos (myod)
100%
80%
60%
40%
20%
0
control
syn4 MO
syn4 MO+syn4 mRNA
control
syn4 MO
syn4 MO+syn4 mRNA
islet1
F
G
H
18/21
20/25
17/22
islet2a
I
J
K
22/23
19/21
21/23

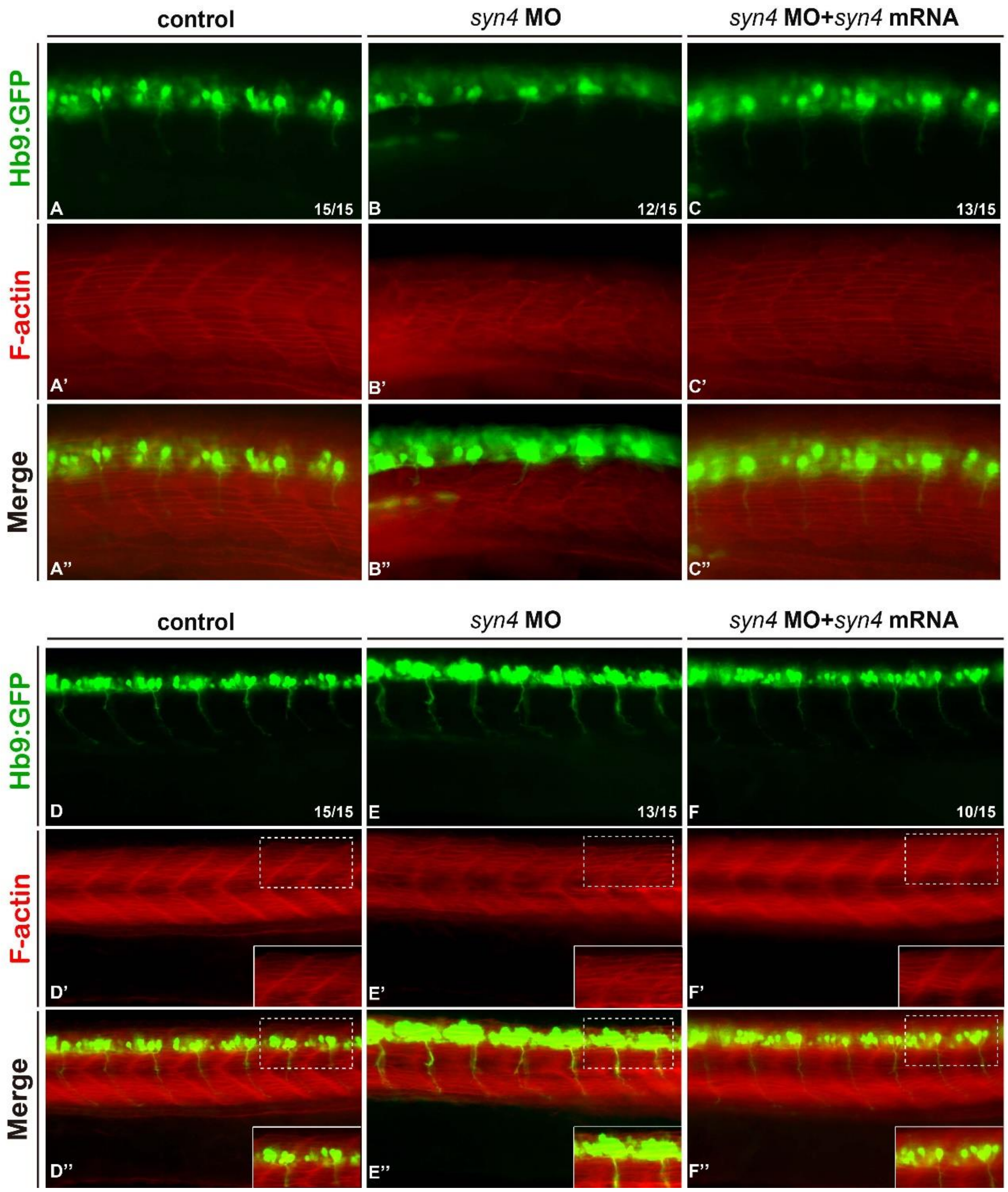
control
syn4 MO
syn4 MO+syn4 mRNA
Hb9:GFP
F-actin
Merge
A
15/15
B
12/15
C
13/15
A'
B'
C'
A''
B''
C''
control
syn4 MO
syn4 MO+syn4 mRNA
Hb9:GFP
F-actin
Merge
D
15/15
E
13/15
F
10/15
D'
E'
F'
D''
E''
F''

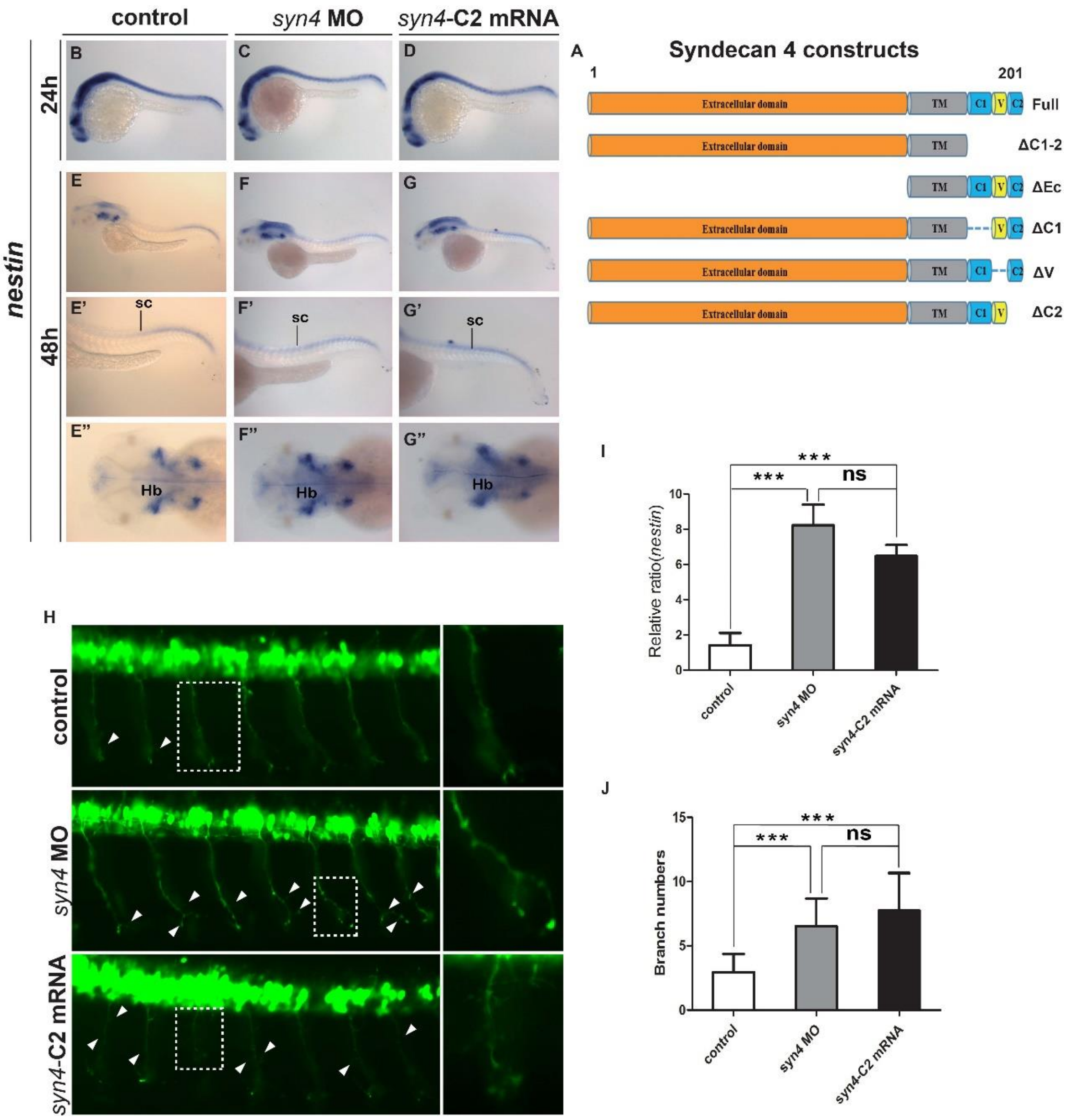
control
syn4 MO
syn4-C2 mRNA
nestin
24h
48h
B
C
D
E
F
G
E'
F'
G'
E''
F''
G''
sc
Hb
A
Syndecan 4 constructs
1
201
Extracellular domain
TM
C1
V
C2
Full
ΔC1-2
ΔEc
ΔC1
ΔV
ΔC2
H
I
Relative ratio(nestin)
***
ns
J
Branch numbers

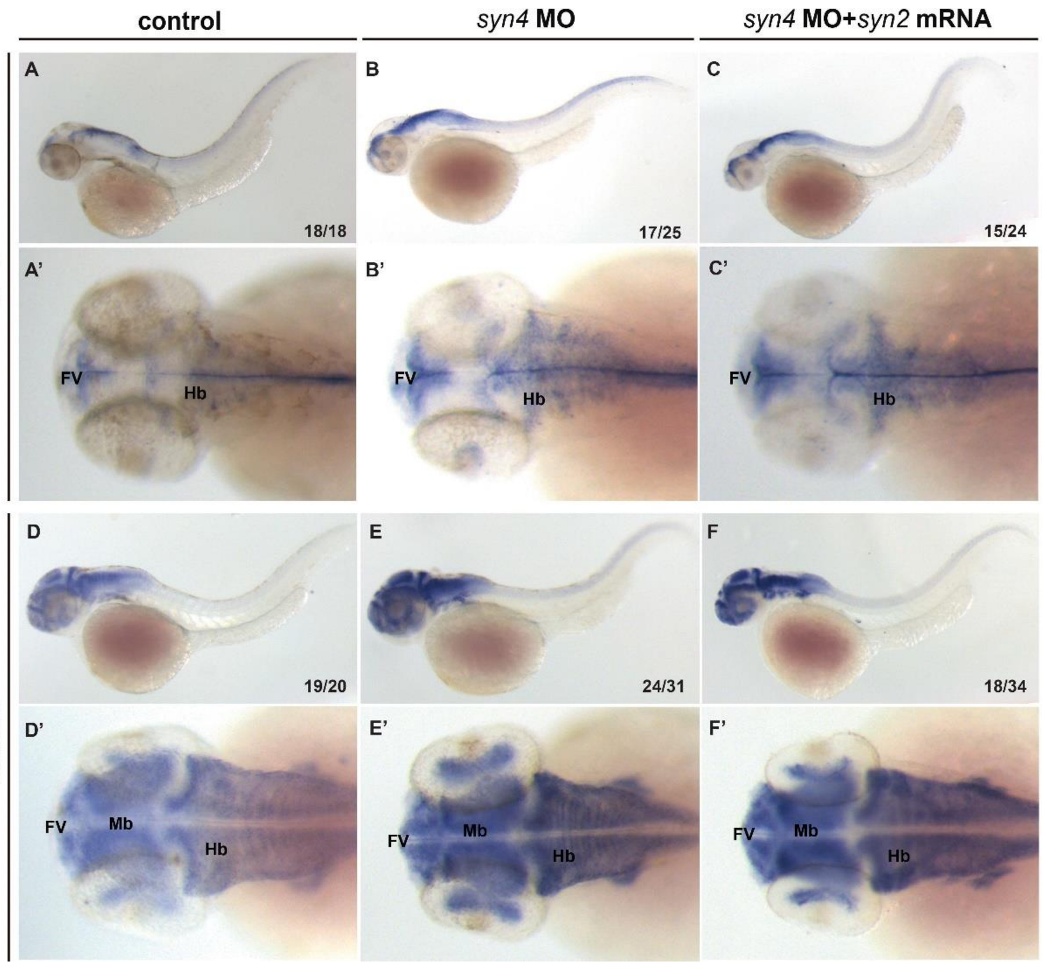
control
syn4 MO
syn4 MO+syn2 mRNA
gfap
elavl3
A
B
C
18/18
17/25
15/24
A'
B'
C'
FV
Hb
D
E
F
19/20
24/31
18/34
D'
E'
F'
Mb

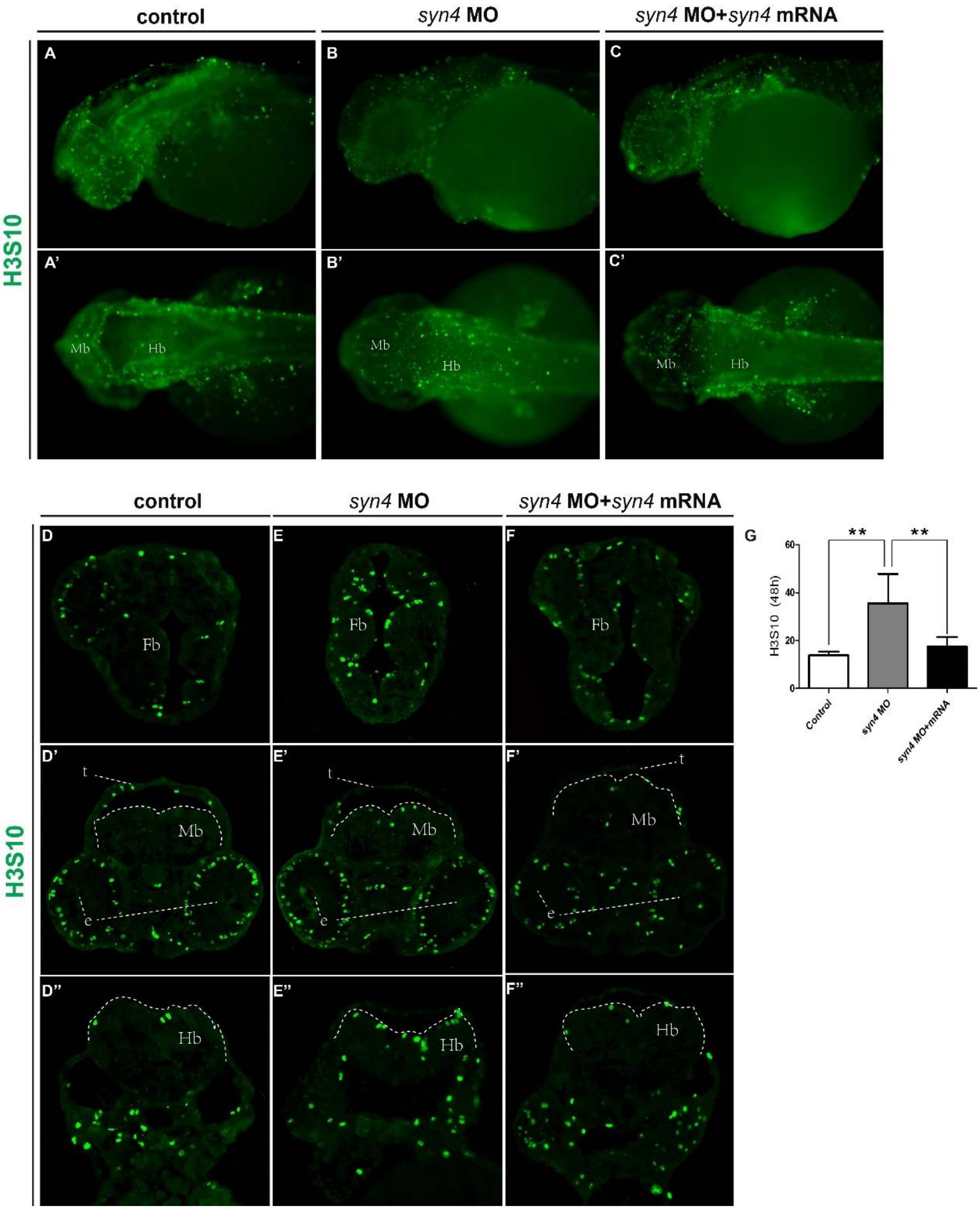
control
syn4 MO
syn4 MO+syn4 mRNA
A
B
C
A'
B'
C'
H3S10
Mb
Hb
D
E
F
D'
E'
F'
D"
E"
F"
Fb
t
e
G
H3S10 (48h)
Control
syn4 MO
syn4 MO+mRNA
**
60
40
20
0

Table S1. Primers for PCR cloning and sqRT-PCR

**Sequence**	**sense-primer**	**antisense-primer**	**Vector**
syn4 coding region (with 5’-UTR)	GGAATTCATTCACTCGGGCTACTTG	CGGATCCCATGCGTAGATTTCTGTGGTTGG	pEGFP-N1
syn4 coding region (without 5’-UTR)	CCGGAATTCATGTTGGTTTTGTCG	CCGCTCGAGTCATGCGTAGATTTC	pcDNA 3.1+
syn2 coding region	CCGGAATTCATGAGGAACTTTTGGAT	TGCTCTAGATTATGCGTAAAACTCCTT	pcDNA 3.1+
syn4-ΔC1	CGCGGATCCATGTTGGTTTTGTCGGCGGCT	TCATTTTTTGTAGATGGGCGTCTTTCCGAGAACGAAGTGAACGAG	pGEM-T easy
	CGCGGATCCATGTTGGTTTTGTCGGCGGCT	TCATGCGTAGATTTCTGTGGTTGGAGCTTTTTTGTAGATGGGCGT	pcDNA 3.1+
syn4-ΔV	CGCGGATCCATGTTGGTTTTGTCGGCGGCT	CCGGAATTCTCATGCGTAGATTTCTGTGGTTGGAGCCAGGTCGTAACTTCCTTC	pcDNA 3.1+
syn4-ΔC2	CGCGGATCCATGTTGGTTTTGTCGGCGGCT	CCGGAATTCTCATTTTTTGTAGATGGGCGT	pcDNA 3.1+
HuC sqRT-PCR	AGCGGAACCAGCCTGCCTAA	TGAGCGTGTTGATAGCCTTGTCG	
gfap sqRT-PCR	TTCGCCAGCTACATCGAGAAGGT	TTGAGAGTGCCGAGGTCTGAGG	
islet1 sqRT-PCR	GCAGCAGCAACCCAACGACAA	TTGAGCCTGGACCACCTTCAGAA	
islet2a sqRT-PCR	CACGGGTCATACGAGTGTGGTTC	TGGTCCAGGTCGCTCTGTAAGG	
olig2 sqRT-PCR	GCAACTCGCTGGAGGAGATGAAG	AAGAGATGCTGCGGTGGAGACT	
slc1a3a sqRT-PCR	ATTATCGCTGTTGATTGGT	ATTTCTTGGTCTCGTTCTC	
Nestin sqRT-PCR	TGGACTGGAGGTGGCAACATACA	AGGCAGGAAGCAGCAGTGGTT	
gapdh sqRT-PCR	AGTTGTAAGCAATGCCTCCTG	CTGGGATGATGTTCTGACTGG	
